# Critical lessons from a pragmatic randomized trial of home-based COVID-19 testing in rural Native American and Latino communities

**DOI:** 10.1111/jrh.12830

**Published:** 2024-03-06

**Authors:** Eliza Webber, Sonia Bishop, Paul K. Drain, Virgil Dupuis, Lorenzo Garza, Charlie Gregor, Laurie Hassell, Geno Ibarra, Larry Kessler, Linda Ko, Alison Lambert, Victoria Lyon, Carly Rowe, Michael Singleton, Matthew Thompson, Teresa Warne, Wendy Westbroek, Alexandra Adams

**Affiliations:** 1 Center for American Indian and Rural Health Equity, Montana State University, Bozeman, Montana, USA; 2 Division of Public Health Sciences, Fred Hutchinson Cancer Research Center, Seattle, Washington, USA; 3 Department of Global Health, University of Washington, Seattle, Washington, USA; 4 Department of Medicine, University of Washington, Seattle, Washington, USA; 5 Department of Epidemiology, University of Washington, Seattle, Washington, USA; 6 Extension Office, Salish Kootenai College, Pablo, Montana, USA; 7 Family and Community Engagement, Sunnyside School District, Sunnyside, Washington, USA; 8 Institute of Translational Health Sciences, University of Washington, Seattle, Washington, USA; 9 Department of Health Systems and Population Health, University of Washington, Seattle, Washington, USA; 10 Providence Medical Research Center, Providence Health Care, Spokane, Washington, USA; 11 Department of Family Medicine, University of Washington, Seattle, Washington, USA

**Keywords:** community-based participatory research, COVID-19, home-based testing, Latino, Native American

## Abstract

**Purpose:**

Native Americans and Latinos have higher COVID-19 infection and mortality rates and may have limited access to diagnostic testing. Home-based testing may improve access to care in rural and underserved populations. This study tests the effect of community health worker (CHW) support on accessibility, feasibility, and completion of COVID-19 home testing among Native American and Latino adults living on the Flathead Reservation in Montana and in Yakima Valley, Washington.

**Methods:**

A two-arm, multisite, pragmatic randomized controlled trial was conducted using block randomization stratified by site and participant age. Active arm participants received CHW assistance with online COVID-19 test kit registration and virtual swabbing support. The passive arm participants received standard-of-care support from the kit vendor. Logistic regression modeled the association between study arm and test completion (primary outcome) and between study arm and test completion with return of valid test results (secondary outcome). Responses to posttest surveys and interviews were summarized using deductive thematic analysis.

**Findings:**

Overall, 63% of participants (*n* = 268) completed COVID-19 tests, and 50% completed tests yielding a valid result. Active arm participants had higher odds of test completion (odds ratio: 1.66, 95% confidence interval [1.01, 2.75]). Differences were most pronounced among adults ≥60 years. Participants cited ease of use and not having to leave home as positive aspects, and transportation and mailing issues as negative aspects of home-based testing.

**Conclusions:**

CHW support led to higher COVID-19 test completion rates, particularly among older adults. Significant testing barriers included language, educational level, rurality, and test kit issues.

## INTRODUCTION

In 2020, the coronavirus disease 2019 (COVID-19) was the third leading cause of mortality in the United States.^1^ Further, the COVID-19 pandemic disproportionately harmed certain historically marginalized populations.^2^ Age-adjusted rates of COVID-19 infection were 1.6× higher among Native Americans and 1.5× higher among Hispanics as compared to non-Hispanic Whites, and Hispanic and Native Americans experienced COVID-19 mortality rates 1.74 and 2.06 times that of non-Hispanic Whites, respectively.^3^

In Montana, the Native American population accounted for 11% of COVID-19 deaths while representing only 6.6% of the state population.^4,5^ This population was particularly impacted during the earlier phases of the COVID-19 pandemic, prior to the availability of vaccines, with Native Americans accounting for 15% of statewide COVID-19 deaths between January 2020 and September 2021.^6^ In Washington state, Hispanics experienced a COVID-19 mortality rate 2-folds higher than the state’s overall COVID-19 mortality rate.^7^ High rates of chronic disease and socioeconomic disparities contribute to these poor health outcomes.^8–14^ Despite high COVID-19 exposure risk, people of color did not have markedly higher testing rates than Whites and were more likely to be positive when tested.^15,16^ Diagnostic testing is a critical tool in reducing the spread of COVID-19 and potentially lessening the disproportionate impact of the disease in Hispanic and Native American communities.

The U.S. Food and Drug Administration (FDA) issued Emergency Use Authorization (EUA) to many COVID-19 tests for home-based testing.^17^ Home-based testing has considerable potential to improve testing access and uptake; however, limited data are available on the acceptance and utility in underserved communities. Nearly half of Native Americans living on tribal land lack access to high-speed internet, and 60% of low-income internet users experience problems with the speed and reliability of their internet.^18,19^ Additionally, lower-income and non-White Americans are less likely to own a computer or smartphone and have lower levels of technology adoption.^19^ Such circumstances may reduce the efficacy of home-based testing.

*Protecting Our Communities* was a randomized controlled trial (RCT), using a community-based participatory research (CBPR) framework to better understand the systems and interventions needed to improve COVID-19 testing in two underserved and vulnerable communities. This study tested the effects of a community health worker (CHW) support model to deliver home-based COVID-19 testing to Hispanics living in Yakima Valley, Washington, and Native Americans living on the Flathead Reservation in Montana. CHW models have proven effective in improving health outcomes, especially among vulnerable populations.^20^ Our study team hypothesized that these communities would have increased home-testing completion rates when testing was made actively available via trusted lay CHWs versus a passively delivered home-testing kit. The trial enabled us to determine best practices and issues with home-based testing delivery within underserved rural Native American and Latino communities.

## METHODS

### Study design

The full methods for the “Protecting Our Community” collaboration model, settings, and study design are published elsewhere.^21^ Briefly, the Center for American Indian and Rural Health Equity at Montana State University (CAIRHE/MSU), University of Washington (UW), Fred Hutchinson Cancer Research Center (Fred Hutch), and Salish Kootenai College (SKC) collaborated with community partners and community advisory boards (CABs) to design and implement a pragmatic RCT, using a mixed-methods approach to include a qualitative substudy on home-based testing experiences. These institutions and communities have long-term academic–community research partnerships.

Hispanic adults residing in the Yakima Valley and Native American adults on the Flathead Reservation were recruited to enroll in the study. Recruitment was conducted through local community coordinators and CHWs using various recruitment strategies, including radio advertisements, word of mouth, community flyers, and social media postings. Those who had previous COVID-19 vaccination and infection were included in our eligibility. Recruitment and enrollment began in April 2021 and concluded in January 2022.

We conducted a single-blinded RCT with participants randomized in a 1:1 ratio by site and age categories (18–25, 26–59, and 60+ years).^22^ All participants received an FDA-approved polymerase chain reaction (PCR) COVID-19 test kit manufactured by Everlywell, to be completed by mailing a self-collected nasal swab to a central laboratory within 14 days of receipt.^23,24^ To mitigate known barriers to home testing for these populations, unrelated to the intervention, such as English language proficiency and access to mail delivery services (UPS, FedEx, etc.), participants in both study arms were provided kit delivery and sample return support coordinated by each site. In addition, graphic, culturally appropriate testing instructions in English and Spanish were added to the commercial kits.

Active arm participants received test completion support from a CHW, while passive arm participants followed the testing kit vendor’s standard of care.^23^ CHW support primarily included assistance with testing kit registration at the onset of the participants enrollment, which was received by all participants in the active study arm. Active arm participants also received as needed support with other testing issues. Passive arm participants were instructed to contact the testing manufacturer customer support if assistance was needed. To maintain long-standing community relations and trust, passive arm participants who reached out directly to CHWs for additional assistance were assisted at the discretion of the CHW. Such instances were documented in our database, and these participants were still included in our analysis, per intent-to-treat protocol.

### Assessment measures

Basic participant demographics including sex, age, primary language, race, and zip code were collected at baseline. Additional demographic information was collected in a follow-up questionnaire, including highest level of education, income, employment status, and health conditions. Participants were also asked to provide feedback on the acceptability and feasibility of home testing in a posttest assessment.

At 14 days postrandomization, we measured the number of test kits “completed” (samples with documented arrival at the testing lab) as our primary outcome, and the number of “successful” tests (i.e., no testing errors) as a secondary outcome. Samples were considered to have testing errors when they were received by the testing lab within 14 days but were unable to be successfully processed because of insufficient sample size, mismatched or missing identifiers, expired samples, or other user errors.

In-depth semistructured interviews were conducted with a subset of 40 participants (*n* = 20 at each site) to collect perceptions of home-based testing and study experiences. Samples were collected using quota sampling to select a convenience sample of 10 test completers and 10 noncompleters at each site location. These quotas were successfully achieved at the Yakima site location but had to be adjusted to a convenience sample at the Flathead location due to smaller sample size and early study termination. Interviews were conducted with 25 test completers and 15 noncompleters. This subsample consisted of 23 participants from the intervention arm and 17 participants from the control arm. Open-ended questions included perception about ease of use or challenges regarding the completion (or incompletion) of their at-home test kit and the helpfulness of their interactions with CHWs.

Participant zip codes were matched to rural–urban commuting area (RUCA) codes to identify participants living in urban and rural areas. Participants living in a metropolitan area (RUCA codes 1.0–3.0) were designated as living in an urban area, while participants living in a micropolitan (RUCA codes 4.0–6.0), small town (RUCA codes 7.0–9.0), or rural area (RUCA code 10.0) were defined as living in a rural area.^25^

### Data analysis

To determine the initial sample size for the study, an absolute increase of 14% was assumed to be a meaningful impact associated with CHW support. Assuming a minimum testing kit completion rate of 70% in the passive study arm, a total sample size of *n* = 400 would provide 90% power to detect an effect size associated with a rate ratio of 1.20, or an absolute difference of 14% (70% in passive, 84% in active). These estimates were derived from prior work with passive strategies, which produced return rates of approximately 80%, in conjunction with study outcome expectations set by CAB members.^26,27^ A futility boundary of 60% was identified by the CABs as justification to prematurely halt trial enrollment if, after completing 25% of expected enrollment, successful test completion fell below that rate.

Descriptive statistics provided overall participant demographic information, stratified by study arm. Results are displayed as numbers and percentages, with Pearson chi-square test being used to assess differences between groups. Simple and multivariable logistic regression modeled the association between testing distribution mechanism (active vs. passive) and completion of self-test within 14 days (yes/no). Multivariable model covariates included age category, community, and sex. Secondary analysis compared active versus passive testing distribution and successful test completion.

All quantitative analyses were conducted using SAS 9.4 (SAS Institute Inc.) with a two-tailed significance level of 0.05.

Open-ended responses from the posttest feedback survey were qualitatively analyzed to identify participants’ top likes and dislikes regarding the home-based testing experience. Senior research team member (LKK) created a preliminary codebook using the in-depth semistructured interview guide. The codebook was used by three data analysts (LT, DvR, TW) to complete deductive coding. An inductive, constant comparison approach was used to identify themes derived from informant discussions beyond the a priori questions.^28^ Themes were compared, and possible linkages across thematic categories were explored. The data were coded using Dedoose, version 9.0.62.^29^ Themes were then validated with the community research teams.

All reported methods and outcomes comply with the 2010 CONSORT reporting guidelines for parallel randomized trials.^30^

## RESULTS

### Enrollment

**FIGURE 1:**
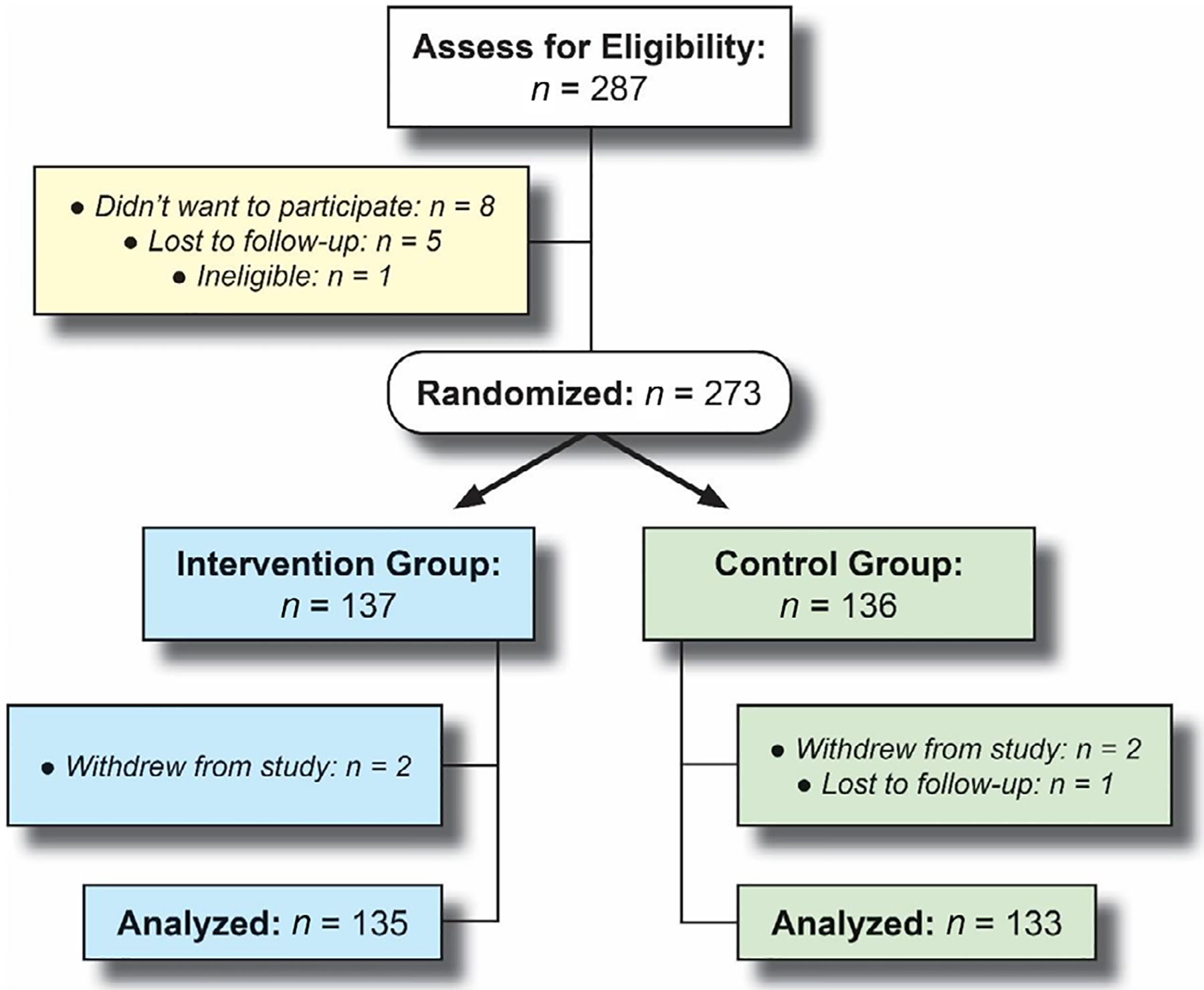
Enrollment and retention flow chart.

A total of 287 individuals were screened for eligibility, 273 of which met eligibility criteria and were randomized to a study arm, and 268 participants were included in the study analysis. Reasons for study exclusion are provided in Figure 1. Recruitment ended after obtaining 68% of our anticipated *n* = 400 sample size due to futility of the testing program. Blinded interim analyses showed test completion rates below our preestablished futility boundary of 60%, providing justification to end the study before reaching our expected sample size.

### Demographics

Home-based testing kits were completed by 169 (63.1%) of the 268 participants, 135 (50.4%) of whom successfully completed the test without sampling errors. Participants were mostly female (80.6%), between the ages of 26 and 59 years (73.5%), Hispanic or Latino (77.2%), and with no education beyond high school (18.3% with a high school degree/GED and 28% with less than a high school degree). Most participants were from the Yakima Valley (74.6%), and most lived in a rural area (89.9%). No statistically significant differences were observed in the demographic distribution of intervention and control group participants (Table 1).

**FIGURE 2:**
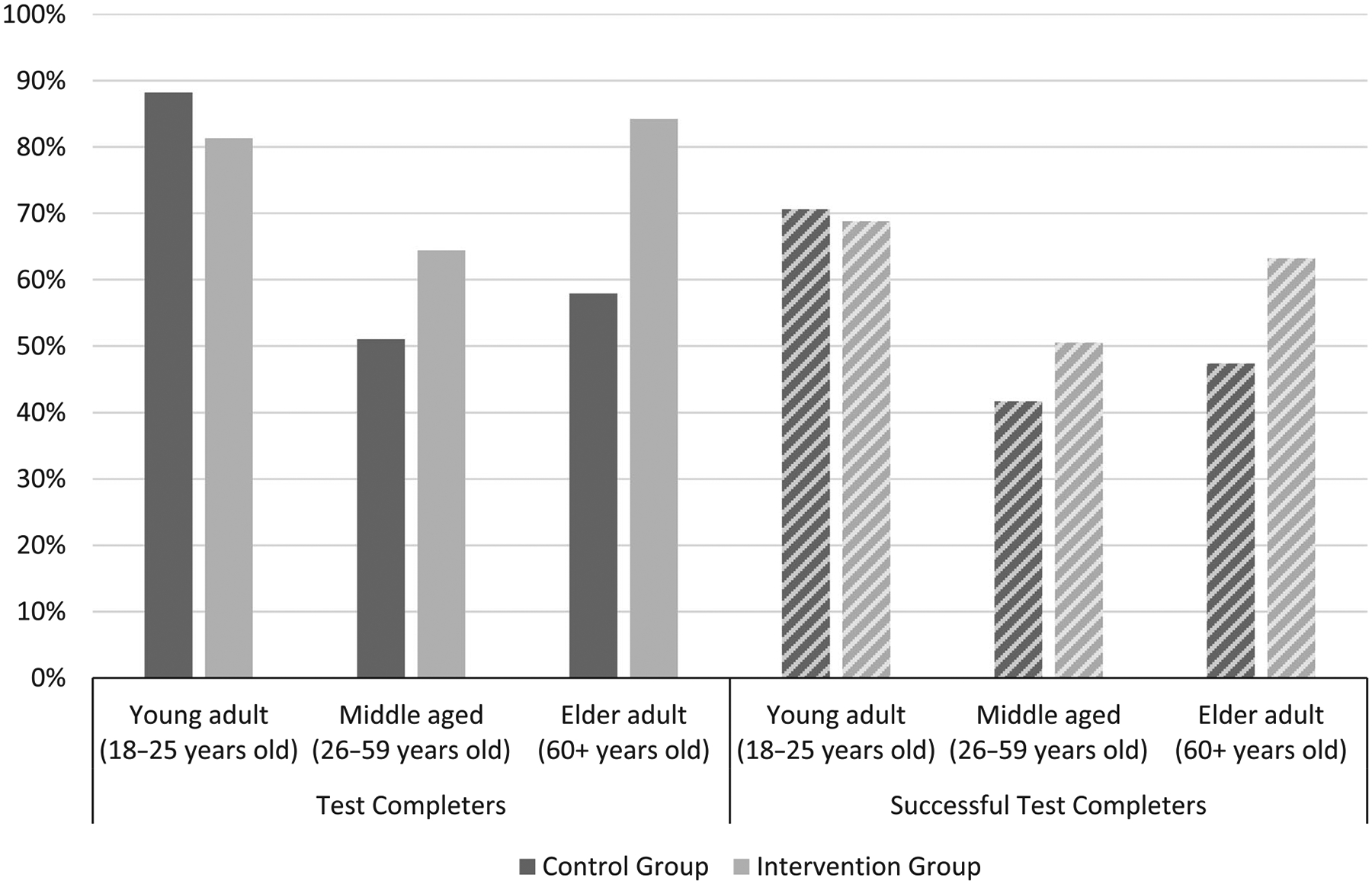
Percent of young, middle aged, and elder adults who completed and successfully completed testing kits, by intervention group.

Test completion rates were highest among young adults (84.9%), with no significant difference between intervention group and control group completion (81.3% vs. 88.2%, *p* = 0.57). Middle-aged adults had the lowest overall test completion rate (57.9%), but completion rates trended higher in the intervention group (64.4%) than the control group (51.0%) (*p* = 0.06). Among elder age adults, test completion rates were 71.1%, with a marginally significant higher percentage of elders in the intervention group (84.2%) completing testing kits than elders in the control group (57.9%) (*p* = 0.07) (Figure 2).

In total, 53 participants experienced testing errors on their first attempt at completing the home-based testing kit, and seven participants received testing errors on both their first and second attempts. Of the 60 total testing errors, the most common types of errors were related to sample expiration (*n* = 28) and incorrect or mismatched identifiers (*n* = 21). Additional sample errors included failure to register kits before sending (*n* = 4), mailing issues (*n* = 4), and improper collection/insufficient sample (*n* = 3).

#### Primary analysis

In an unadjusted logistic regression model, participants in the active study arm had 1.79 higher odds of completing the home-based testing kit compared to those in the passive study arm (95% confidence interval [CI] [1.03, 2.80], *p* = 0.04). A borderline significant association remained after controlling for differences in age category, sex, and site location (odds ratio [OR]: 1.70, 95% CI [1.00, 2.88], *p* = 0.05). No statistically significant association was observed between active versus passive study arm and successful test completion (unadjusted OR: 1.39, 95% CI [0.86, 2.26], *p* = 0.18; adjusted OR: 1.34, 95% CI [0.82, 2.19], *p* = 0.25) (Table 2).

#### Qualitative analysis

The posttest feedback survey was completed by 165 of test-completing participants. A total of 42% of these participants thought the home-based testing kits were “easy to use,” with “clear instructions” (*n* = 15) and “helpful video instructions” (*n* = 2) specified as reasons for the ease of use. “Convenience” was mentioned by 28% of respondents, with “not having to leave home” (*n* = 18), “able to do on own time” (*n* = 6), and “convenient return of results” (*n* = 3) specified as reasons for the convenience. The most common dislike participants had was “coordinating transportation logistics to mail samples to testing lab,” which was cited by 18% of respondents, with “Dropbox/UPS mailing location too far away” (*n* = 15) and “lack of transportation” (*n* = 6) specified as the top reasons for disliking this testing aspect. Participants also disliked “Long/overwhelming instructions” (13%) and “experiencing sample errors” (10%) (Table 3).

Results from the in-depth interviews echoed the responses received in the posttest feedback survey, with three major themes noted (Table 4).

### Study and test facilitators

Most participants reported that the test was easy to complete, with clear instructions, communication from staff to answer questions, and the convenience of at-home testing compared to in-person facilities cited as examples. Two individuals from the Yakima site location noted that having instructions in Spanish was a motivator due to the high portion of bilingual populations in their community.

### Study and test barriers

A large majority of participants shared that UPS drop-off sites were a barrier in the study. For example, UPS drop-off locations were few and far between, which made it difficult to find and drive to a location. Interviewees also described difficulty accessing UPS because of operating hours conflicting with their work schedules.

Being too busy was the third most reported barrier by participants, making it difficult to complete and ultimately return the kit to the UPS store in a timely fashion.

Lastly, figuring out how to complete the test kit was the fourth most reported barrier with some individuals describing the instructions confusing, unclear, and/or too long to read.

### Experience with CHWs

Two thirds of participants stated the CHWs were a positive addition to the study and made the process easier to complete the kit.

Interviewees shared how they appreciated being able to ask questions to the CHW, how the CHW could walk them through the test process, the reminder calls CHWs would provide, and the close contact participants felt they could have with a CHW.

However, one third of participants shared they had no or very little interaction with a CHW, and five participants stated how they would have liked additional assistance from the CHWs. The majority (4/5) of those who would have liked additional help were randomized to the passive arm of the study and therefore did not receive CHW support.

## DISCUSSION

### Main findings

In these rural and underserved communities, the overall rate of test completion with valid results was 50%, well below our a priori assumptions prior to the study start. However, the trusted CHW support significantly improved test completion rates and was particularly beneficial to middle age and older adults who, compared to young adults, are more burdened by time restrictions and limited technology literacy, respectively. While CHWs may facilitate test completion, existing sociocultural and infrastructure barriers limit the efficacy and viability of home-based, mail-in testing in these communities. Anecdotally, Yakima community public health nurses reported a less than 50% completion rate for their patients when using the Everlywell tests during the first year of the pandemic, even with support.

Many Yakima participants had limited formal education, with 16% attaining an elementary school education or less. This may have contributed to participants feeling overwhelmed by all the testing instructions and reading materials, as noted in our in-depth interview themes. Language barriers, with 41% of participants in Yakima speaking Spanish as a primary language, also may have contributed to confusion and difficulty since certain customer support components were only offered by the testing manufacturer in English, including the videos.

The rurality of the Flathead site location, with 94% of participants living in small towns or rural areas, presented unique challenges, particularly with regard to transportation and access to postal services. Participants described having to drive up to 40 miles to the nearest overnight shipping location. This inconvenience is amplified by the limited operating hours of postal service facilities, which often overlap with participants’ work hours. Public transportation is often limited or entirely unavailable in rural areas, requiring participants to own or have access to motor vehicles and the ability to safely operate and afford the upkeep and operational costs of them. Yet, 10% of Native American households lack access to a vehicle, compared to just 6% of White households.^31^

Native American and Hispanic cultures both often embrace long full names, which would be difficult to fit on the sample tubes provided by the testing manufacturer. This likely contributed to testing errors caused by mismatched identifiers. Such errors were equally prominent at both the Yakima Valley and Flathead Reservation site locations.

Identifying solutions to these testing barriers is beyond the scope of this study; however, initial steps may include offering instructions written at a lower reading level, accurately translated to non-English languages, with diverse and racially inclusive images. To properly address these evident barriers to home-based testing, future testing manufacturers must ensure that their product is thoroughly vetted with individuals from a diverse range of socioeconomic backgrounds, especially those representing lower literacy levels, limited technology access and proficiency, non-English speakers, minority races and ethnicities, and rural environments.

### Comparison with existing literature

While there is robust evidence that individuals can obtain the needed samples themselves (e.g., nasal, saliva) for COVID-19 with similar accuracy to clinicians, leading to FDA EUA for several self-collected sampling test manufacturers, there are multiple other barriers to accessing and conducting tests.^32–34^ To our knowledge, this is the first study to examine the efficacy of home-based COVID-19 self-testing kits by test completion rate. The low rates of test completion in our study contrast with higher completion rates among other types of self-collection test kits, such as HIV self-tests, which have been shown to have high efficacy in similarly marginalized communities.^35^ This discrepancy in home-testing outcomes is likely to be partially attributed to differences in the health conditions being tested, which may affect individual perceptions surrounding risk and severity of infection and benefits and barriers to testing, among other complex decision-making factors. Commercially available home-based tests also vary widely in sample collection and testing mechanisms. The prior studies on home-based HIV testing all used oral swab samples, which may be easier to collect than nasal swab samples, and provided rapid results without needing to mail the samples. These factors are likely to influence home-testing completion rates.

With the approval and authorization by the FDA of multiple COVID-19 tests, including tests that are completed entirely at home, as well as those that involve returning a sample to a central lab for testing, it is important to understand how communities who most need these tests can best be served.

### Strengths and limitations

This study provides novel information regarding the efficacy of a home-based PCR testing for COVID-19 in rural minority communities. Notable strengths of the study can be summarized as follows. (1) Using a robust RCT design provides unbiased and scientifically valid evidence supporting the benefits of active assistance from CHWs on home-testing completion rates. (2) Results fill critical gaps in knowledge needed to inform future diagnostic testing efforts in non-English speaking communities, those with limited digital literacy or internet access, advanced age or health conditions impacting ability to administer the test (e.g., dexterity to self-swab, ability to see instructions), and rural communities with limited access to transportation. (3) All study protocols and materials were developed with input and approval from CABs, ensuring that methods and materials were culturally appropriate and in line with community needs and values. Additionally, (4) the study’s mixed method approach provides rich qualitative data to help explain the reasons behind low test-completion rates, allowing for a more targeted response to improving future testing models.

This study had several limitations. (1) The availability of home-based COVID-19 testing kits was limited, and rapid antigen tests were not yet widely available when our clinical trial opened for enrollment in spring of 2021. As such, we faced challenges identifying and acquiring a testing kit that would meet our needs. (2) The testing kit manufacturer used in our study provided participants with autogenerated email and text updates about test results; however, these communications were only delivered in English. This presented a language barrier to our Spanish-speaking participants and is an evident flaw of most commercially available testing kits that prioritize English language for initial implementation. (3) We were unable to examine the immediate impact of test results on actions by tested individuals, since samples needed to be returned to a central laboratory for analysis. Our findings therefore may not apply to emerging tests that can be completed entirely at home. (4) Because completion rates fell below our preestablished futility boundary of 60%, the study was stopped early before reaching full enrollment, thus limiting our statistical power. This particularly limited our ability to conduct sensitivity analyses assessing the interactive effects of various demographic factors and CHW support on test completion. However, our sample size provided sufficient power to detect statistical significance in our primary analysis, allowing us to provide valuable insight into the body of literature on home-based COVID-19 testing in underserved communities.

## CONCLUSION

Our results suggest that rural and underserved communities face many barriers to using home-based testing. However, the use of such testing may still be a viable option if manufacturers can better account for socioeconomic differences and overcome limitations related to literacy, language, and rurality in product development. CHW-supported home testing improved uptake, particularly among older participants in our study. As such, health care providers should consider partnering with community health programs to offer home-testing support and improve testing efficacy. This strategy may prove useful in future pandemics or in situations where home testing could be used for other conditions and would serve to reduce the burden on local health care providers, particularly in rural areas. Results from the “Protecting Our Community” trial provide critically needed insight into the feasibility of home-based testing and unique needs that must be addressed to reduce testing disparities and improve health outcomes in underserved rural communities. These findings may have widespread impact as they can help inform health policies and services on both a local and a national scale.

## Figures and Tables

**TABLE 1:** Participant demographics, by study arm.

			Study arm		
		Overall (*N* = 268)	Control group (*N* = 132)	Intervention group (*n* = 136)	*p*-value
Sex					
Male		49 (18.3%)	22 (16.7%)	27 (19.9%)	0.45
Female		216 (80.6%)	110 (83.3%)	106 (77.9%)	
Missing		3 (1.1%)	0 (0.0%)	3 (2.2%)	
Age category					
18–25 years old		33 (12.3%)	17 (12.9%)	16 (11.8%)	0.95
26–59 years old		197 (73.5%)	96 (72.7%)	101 (74.3%)	
60+ years old		38 (14.2%)	19 (14.4%)	19(14.0%)	
Location					
Flathead Reservation		68 (25.4%)	33 (25.0%)	35 (25.7%)	0.89
Yakima Valley		200 (74.6%)	99 (75.0%)	101 (74.3%)	
Race/Ethnicity					
Native American or Alaska Native		60 (22.4%)	28 (21.2%)	32 (23.5%)	0.65
Hispanic or Latino		207 (77.2%)	104 (78.8%)	103 (76.3%)	0.63
Non-Hispanic White		18 (6.7%)	9 (6.8%)	9 (6.6%)	0.95
Education					
No formal education		4 (1.5%)	2 (1.5%)	2 (1.5%)	0.88
Elementary school		28 (10.5%)	14 (10.6%)	14 (10.3%)	
Some high school		43 (16.0%)	19 (14.4%)	24 (17.7%)	
High school degree or GED		49 (18.3%)	28 (21.2%)	21 (15.4%)	
Tech school		9 (3.4%)	4 (3.0%)	5 (3.7%)	
Some college		54 (20.2%)	28 (21.2%)	26 (19,1%)	
College degree		32 (11.9%)	13 (9.9%)	19 (14.0%)	
Graduate school degree		5 (1.9%)	2 (1.5%)	3 (2.2%)	
Missing		44 (16.4%)	22 (16.7%)	22 (16.2%)	
Rural–urban commuting area (RUCA)					
Urban	Metropolitan	27 (10.1%)	17 (12.9%)	10 (7.4%)	0.22
	Micropolitan	165 (61.6%)	77 (58.3%)	88 (64.7%)	
Rural					
	Small town	36 (13.4%)	21 (15.9%)	15 (11.0%)	
	Rural	40 (14.9%)	17 (12.9%)	23 (16.9%)	

**TABLE 2:** Association between receiving community health worker support and test completion.

		Unadjusted model			Multivariate model^a^		
Outcome variable	Predictor variable	Odds ratio	95% confidence interval	*p*-value	Odds ratio	95% confidence interval	*p*-value
Test completion		1.79	[1.03, 2.80]	0.04	1.70	[1.00, 2.88]	0.05
Successful test completion	Intervention group (active vs. passive)	1.39	[0.86, 2.26]	0.18	1.34	[0.82, 2.19]	0.25

a Adjusting for age category, sex, and site location.

**TABLE 3:** Most frequently cited positive and negative aspects of home-based testing kits.

Theme	*N*	%	Subtheme	*N*	%
**Positive aspects**					
Easy to use	69	42%	Clear instructions	15	9%
			Video instructions were helpful	2	1%
Convenience	46	28%	Not having to leave home	18	11%
			Able to do on my own time	6	4%
			Convenient return of results	3	2%
			Return label was included in kit	1	1%
			No need for refrigeration	1	1%
Not having to leave home	40	24%	No contact/exposure to others	11	7%
			More comforting environment than going to public testing center	7	4%
Everything	22	13%			
Fast	12	7%			
Didn’t cause pain or discomfort	10	6%			
Privacy	8	5%			
Able to do it by myself	4	2%			
Sanitary	3	2%			
Negative aspects					
Nothing	81	49%			
Coordinating transportation logistics to mail samples to testing lab	30	18%	Dropbox/UPS too far away	15	9%
			Lack of transportation/vehicle	6	4%
			Cost of gas	1	1%
			Difficulty finding nearest UPS location	1	1%
			Outdated UPS drop-off location pamphlet	1	1%
Long/overwhelming instructions	21	13%	Excessive/repetitive	3	2%
			Too much POC information included in testing kit	1	1%
Experiencing sample errors	16	10%	Didn’t receive a test result	11	7%
			Had to take the test twice	2	1%
			Not knowingwhat wentwrong	1	1%
Unclear instructions	8	5%			
Time consuming	6	4%			
Didn’t like nasal swab	6	4%	Sample collection was uncomfortable	2	1%
Long wait for return of results	7	4%			
Difficulty with the technology requirements	4	2%			

**TABLE 4:** In-depth interviews content analysis.

Code	Applied	*Illustrative Quotes*
Study and Test Facilitators: Discussions around existing programs, efforts, and resources for COVID-19 testing (i.e., information, transportation, etc.)	158	*“You know, it was fairly easy. It was convenient… … I got my tests quickly. I got my test done conveniently and it didn’t hurt or anything*.” *“It was a good experience, it was nice to know that there is alternative to having to go to a doctor’s office to get testing done. Especially for instances where like you’re just exposed but you don’t have symptoms, but you don’t want to get anybody else sick. That’s like the perfect scenario to be able to just test at home*.”
Study and Test Barriers: Discussions around what made it difficult to participate in the study and/or complete the study	96	*“My big challenge was just like, I don’t go anywhere, I have a gas guzzler, and the only places were 30 miles or 30 minutes, one way and an hour the other way… … and then it was confusing, too, because I’m like, OK, so drop it off either. Monday, Tuesday, Wednesday. And then Thursday is tricky, and then it wasn’t in my realm of remembering things until it was. And then when it was, then I did it wrong and I dropped it off. Well, thank God it went through. That was great. Yeah. And so, yeah, I mean that was my only hang up was just getting it to the [UPS], taking the test was no issue, just getting it to the UPS was the only issue.”* *“The window was a little difficult for me, just in the sense of I’m kind of tied down with my particular work, and I only have a half hour for lunch. So it was just getting that stuff done in a timely fashion to get over there because I get constant interruptions at work.”* *“When I got the kit, I read through it and I knew I had a certain amount of time to get it done. And then, of course, I got busy. And I think it even sat for a couple of days. And then I realized, holy crap, I got to get this done.”* *“A lot of reading [referring to the flyers and brochures inside the testing kit], and it would be nice if it was just like, just test it and send it in. Something quick*.’
Experience with Community Health Workers: Discussion about the adequacy of community health worker support received by participants in the study	62	*“Yeah, they’re really good. They were really good about calling me back. Telling me where to do the drop-off, you know, if I needed it?… … Were really quick about telling me when my package was going to arrive. Yeah, it was super easy, I mean, they ship it straight to your house, it was cool. I liked it. I like the study. Yeah, they told us when they expect our package. And then other than that, if we had questions, just reach out. But I didn’t have any. So like pretty much the pamphlet says it all. It tells you literally everything to do. So, yeah, a lot of thought went into that.”* *“I felt like it was very little, Like, for a minute, I vaguely remember who they are, at least the ones here. And then I think I had like, that woman who told me my results. And I was thankful for that. But there really wasn’t any [further help] once I had signed up. Because, like, part of me even felt like even a little reminder, like hey did you turn your test in… … but there was nothing until I got my results.”*

## References

[1] Ahmad FB, Anderson RN. The leading causes of death in the US for 2020. JAMA. 2021;325(18):1829–1830. 33787821 10.1001/jama.2021.5469 PMC8145781

[2] Lopez L, Hart LH, Katz MH. Racial and ethnic health disparities related to COVID-19. JAMA. 2021;325(8):719–720. 33480972 10.1001/jama.2020.26443

[3] Provisional COVID-19 deaths by race and Hispanic origin, and age. Centers for Disease Control and Prevention. Accessed September 16, 2022. https://data.cdc.gov/d/ks3g-spdg

[4] MT Department of Health and Human Services. Demographic Information for COVID Cases, Montana. Accessed September 9, 2022. https://dphhs.mt.gov/assets/publichealth/CDEpi/DiseasesAtoZ/2019-nCoV/EpiProfile/DemographicTables20220909.pdf

[5] US Census. QuickFacts Montana. Accessed September 16, 2022. https://www.census.gov/quickfacts/MT

[6] Koch T, Williamson L. Covid-19 Associated Deaths among Montana Residents, Provisional Data January 2020–September 2021. Montana Department of Public Health and Human Services (DPHHS); 2021.

[7] Washington State Department of Health. COVID-19 Morbidity and Mortality by Race, Ethnicity and Spoken Language in Washington State. Washington State Department of Health; 2022.

[8] Centers for Disease Control and Prevention. Public Health Information for Tribes. Accessed September 16, 2022. https://www.cdc.gov/tribal/data-resources/information/chronic-diseases.html

[9] Clerkin KJ, Fried JA, Raikhelkar J, COVID-19 and cardiovascular disease. Circulation. 2020;141(20):1648–1655. 32200663 10.1161/CIRCULATIONAHA.120.046941

[10] Kaiser Family Foundation. COVID-19 Presents Significant Risks for American Indian and Alaska Native People. Kaiser Family Foundation; 2020.

[11] Calo WA, Murray A, Francis E, Bermudez M, Kraschnewski J. Peer reviewed: reaching the Hispanic community about COVID-19 through existing chronic disease prevention programs. Prev Chronic Dis. 2020;17:E49. 32584753 10.5888/pcd17.200165 PMC7316420

[12] Richardson S, Hirsch JS, Narasimhan M, Presenting characteristics, comorbidities, and outcomes among 5700 patients hospitalized with COVID-19 in the New York City area. JAMA. 2020;323(20):2052–2059. 32320003 10.1001/jama.2020.6775 PMC7177629

[13] Centers for Disease Control and Prevention. Health Equity. Accessed September 20, 2022. https://www.cdc.gov/healthequity/whatis/?CDC_AA_refVal=%3A%2F%2Fwww.cdc.gov%2Fcoronavirus%2F2019-ncov%2Fcommunity%2Fhealth-equity%2Frace-ethnicity.html

[14] Tai DBG, Shah A, Doubeni CA, Sia IG, Wieland ML. The disproportionate impact of COVID-19 on racial and ethnic minorities in the United States. Clin Infect Dis. 2020;72(4):703–706. 10.1093/cid/ciaa815 PMC7337626 32562416

[15] McCormack G, Avery C, Spitzer AK-L, Chandra A. Economic vulnerability of households with essential workers. JAMA. 2020;324(4):388–390. 32556217 10.1001/jama.2020.11366 PMC7303901

[16] Rubin-Miller L, Alban C, Artiga S, Sullivan S. COVID-19 racial disparities in testing, infection, hospitalization, and death: analysis of epic patient data. Kaiser Family Foundation; 2020.

[17] US Food and Drug Administration. In Vitro Diagnostics EUAs. Accessed September 20, 2022. https://www.fda.gov/medical-devices/coronavirus-disease-2019-covid-19-emergency-use-authorizations-medical-devices/in-vitro-diagnostics-euas

[18] Martin M For the First Time, Census Bureau Data Show Impact of Geography, Income on Broadband Internet Access. US Census; 2018. Accessed September 20, 2022. https://www.census.gov/library/stories/2018/12/rural-and-lower-income-counties-lag-nation-internet-subscription.html

[19] Vogels E Digital Divide Persists Even as Americans with Lower Incomes Make Gains in Tech Adoption. Pew Research Center; 2021.

[20] Kim K, Choi JS, Choi E, Effects of community-based health worker interventions to improve chronic disease management and care among vulnerable populations: a systematic review. Am J Public Health. 2016;106(4):e3–e28. 10.2105/AJPH.2015.302987 PMC4785041 26890177

[21] Thompson MJ, Drain PK, Gregor CE, A pragmatic randomized trial of home-based testing for COVID-19 in rural Native American and Latino communities: protocol for the “Protecting our Communities” study. Contemp Clin Trials. 2022;119:106820. 35691487 10.1016/j.cct.2022.106820 PMC9181367

[22] Harris PA, Taylor R, Thielke R, Payne J, Gonzalez N, Conde JG. Research electronic data capture (REDCap)—a metadata-driven methodology and workflow process for providing translational research informatics support. J Biomed Inform. 2009;42(2):377–381. 18929686 10.1016/j.jbi.2008.08.010 PMC2700030

[23] Everlywell. COVID-19 Test Home Collection Kit DTC. Accessed December 20, 2022 https://www.everlywell.com/products/covid-19-test/?g_network=g&g_adid=626540580471&g_keyword=everlywell%20covid%2019%20test%20home%20collection%20kit&g_adtype=search&g_campaign=CAC+-+COVID-19+Test+Home+Collection+Kit+-+Target+ROAS+Bidding&g_keywordid=kwd-931665130313&g_acctid=652-099-8287&g_campaignid=12199387356&g_adgroupid=120024815471&utm_source=google&utm_medium=cpc&utm_campaign=covid&gclid=Cj0KCQiA14WdBhD8ARIsANao07jOhwFVJ_PL8sgKIHvhwAQTEOcf7Um_qZSZ7hNEI6Fn2uNkw7S6-PEaAgXnEALw_wcB

[24] Etherington D Everlywell gains first FDA authorization for a standalone, at-home, COVID-19 test sample collection kit. Accessed April 20, 2023 https://techcrunch.com/2020/05/16/everylwell-gains-first-fda-authorization-for-a-standalone-at-home-covid-19-test-sample-collection-kit/

[25] US Department of Agriculture Economic Research Service. 2010 Rural-Urban Commuting Area (RUCA) Codes. Accessed January 19, 2024 https://www.ers.usda.gov/data-products/rural-urban-commuting-area-codes/documentation/

[26] Kim AE, Brandstetter E, Wilcox N, Evaluating specimen quality and results from a community-wide, home-based respiratory surveillance study. J Clin Microbiol. 2021;59(5):e02934–20 33563599 10.1128/JCM.02934-20 PMC8091861

[27] Geyer RE, Kotnik JH, Lyon V, Diagnostic accuracy of an at-home, rapid self-test for influenza: prospective comparative accuracy study. JMIR Public Health Surveill. 2022;8(2):e28268. 35191852 10.2196/28268 PMC8905479

[28] Miles M, Huberman M, Saldana J. Qualitative Data Analysis: A Methods Sourcebook. 4th ed. SAGE; 2020.

[29] Dedoose Version 9.0.62: web application for managing, analyzing, and presenting qualitative and mixed method research data. SocioCultural Research Consultants, LLC; 2022.

[30] Schulz KF, Altman DG, Moher D. CONSORT 2010 statement: updated guidelines for reporting parallel group randomised trials. J Pharmacol Pharmacother. 2010;1(2):100–107. 21350618 10.4103/0976-500X.72352 PMC3043330

[31] 2021: ACS 1-Year Estimates Selected Population Profiles. US Census. Accessed March 10, 2023 https://data.census.gov/table?g=0100000US&y=2021

[32] Dharanikota S, LeRouge CM, Lyon V, Durneva P, Thompson M. Identifying enablers of participant engagement in clinical trials of consumer health technologies: qualitative study of influenza home testing. J Med Internet Res. 2021;23(9):e26869. 34519664 10.2196/26869 PMC8479603

[33] Waghmare A, Krantz EM, Baral S, Reliability of self-sampling for accurate assessment of respiratory virus viral and immunologic kinetics. J Infect Dis. 2022;226(2):278–286. 32710762 10.1093/infdis/jiaa451 PMC7454707

[34] Wang ML, Behrman P, Dulin A, Addressing inequities in COVID-19 morbidity and mortality: research and policy recommendations. Transl Behav Med. 2020;10(3):516–519. 32542349 10.1093/tbm/ibaa055 PMC7337775

[35] Johnson CC, Kennedy C, Fonner V, Examining the effects of HIV self-testing compared to standard HIV testing services: a systematic review and meta-analysis. J Int AIDS Soc. 2017;20(1): 21594. 28530049 10.7448/IAS.20.1.21594 PMC5515051

